# A validated LC-MS/MS method for cellular thyroid hormone metabolism: Uptake and turnover of mono-iodinated thyroid hormone metabolites by PCCL3 thyrocytes

**DOI:** 10.1371/journal.pone.0183482

**Published:** 2017-08-24

**Authors:** Keith H. Richards, Nancy Schanze, Ray Monk, Eddy Rijntjes, Daniel Rathmann, Josef Köhrle

**Affiliations:** 1 Charité-Universitätsmedizin Berlin, Institut für Experimentelle Endokrinologie, Augustenburger Platz 1, Berlin, Germany; 2 Chromicent GmbH, Johann-Hittorf-Strasse 8, Berlin, Germany; University Claude Bernard Lyon 1, FRANCE

## Abstract

Tyrosine and phenolic ring de-iodination of thyroid hormones (TH) is crucial for regulating their physiological activity. Furthermore, reactions such as de-carboxylation to thyronamines (TAM) and de-amination to thyroacetic acids (TAc) produce TH metabolites (THM) with distinct biological properties. This needs to be considered when studying effects of TH and THM. The accurate and precise quantitative analysis of TH and THM in cell culture supernatants and cell lysates are key procedures required for studying the *in vitro* metabolism of TH. We report here the development of a liquid-liquid extraction/isotope dilution-liquid chromatography-electrospray tandem mass spectrometry (LC-MS/MS) method for the quantification of 9 thyronines (TN) and 6 TAM in human hepatocellular carcinoma Hep G2 cell lysate extracts. In addition, we adapted the method to quantify TH, TAM and TAc, in cell lysates of FBS-depleted rat thyroid epithelium PCCL3 cells. The methods for both cell lines were validated by rigorous assessment of linearity, limits of quantification and detection (LLOQ and LLOD respectively), intra- and inter-day accuracy, precision, process efficiency (PE), matrix effect (ME) and relative recovery (RE). Calibration curves covering 11 concentrations (based on 400 μl of lysate) were linear in the range 0.016–50 nM and 0.010–50 nM for Hep G2 and PCCL3 cells respectively. The lower limits of quantification were in the range 0.031 to 1 nM. We applied the PCCL3 version of the LC-MS/MS method to the analysis of lysed cell extracts from PCCL3 cells that had been incubated with 3-iodo-L-thyronine (T_1_), 3-iodothyronamine (3-T_1_AM) and 3-iodothyroacetic acid (3-T_1_Ac). Over the course of 30 minutes incubation 3-T_1_AM was de-iodinated to 4-[4-(2-aminoethylphenoxy)]phenol (thyronamine, T_0_AM) and de-aminated to 3-T_1_Ac respectively, whilst T_1_ underwent de-iodination to T_0_. This data indicates avid metabolism of these mono-iodinated compounds and the utility of LC-MS/MS to quantify such cellular metabolism.

## Introduction

**Fig 1:**
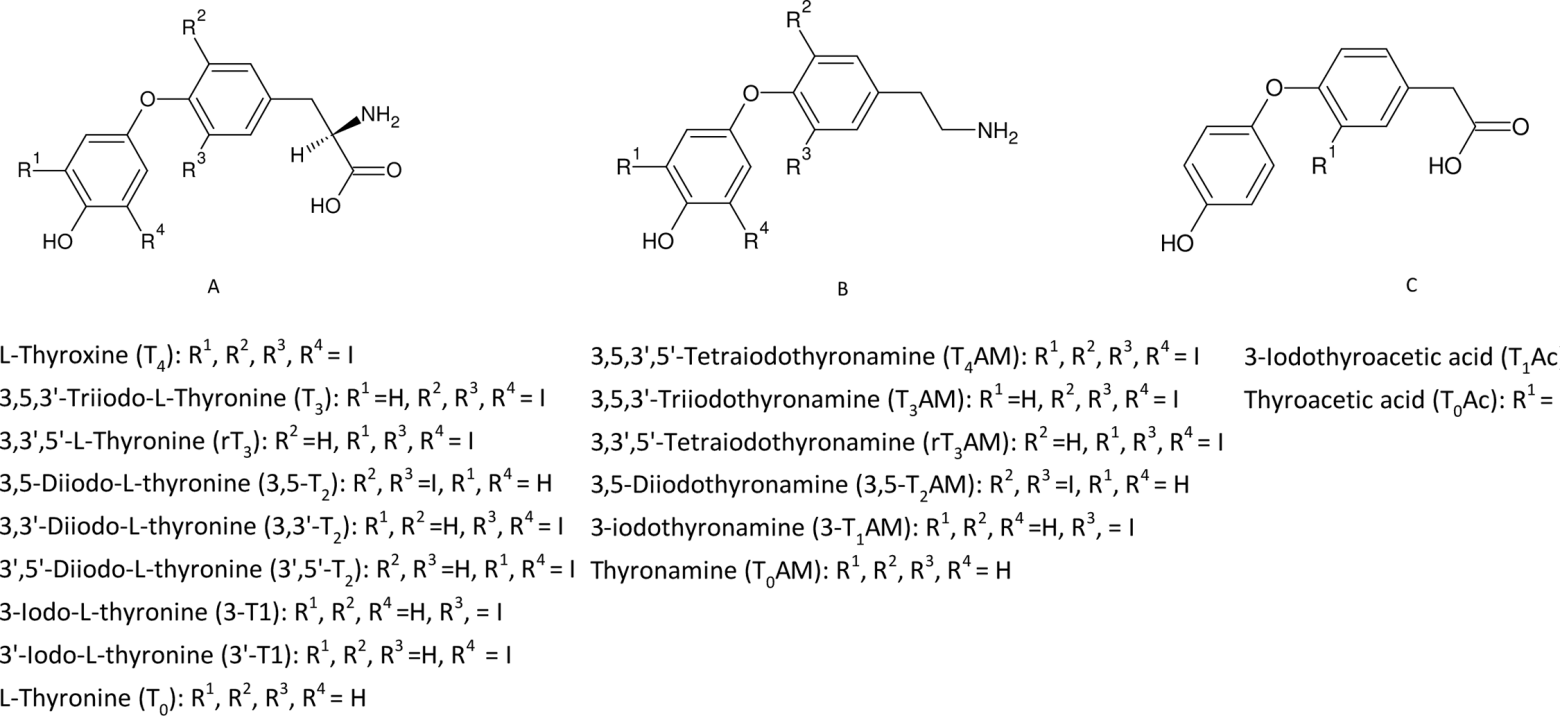
Molecular structures of TH, TAM and TAc.

The thyroid hormone (TH) 3,5,3’-triiodo-L-thyronine (T_3_) regulates a variety of processes that ensure proper development, growth and metabolism. Most of the circulating T_3_ is generated by de-iodination of the phenolic ring of the less active TH 3,5,3’,5’-tetraiodo-L-thyronine (T_4_)–a reaction catalysed by deiodinases 1 and 2 *in vivo* [1, 2]. Inactivation of T_4_ is also accomplished by de-iodination, and leads to the formation of 3,3’,5’-triiodothyronine (reverse T_3_, rT_3_); similarly, de-iodination of T_3_ generates either the active 3,5-diodothyronine (3,5-T2) or the inactive 3,3’-diodothyronine 3,3’-T_2_), [3]. Besides de-iodination reactions, several other pathways of TH metabolism are possible. TH metabolites (THM, see Fig 1.) include thyronamines (TAM), resulting from TH de-carboxylation, and thyroacetic acids (TAc) resulting from the deamination of TAM. Some of these THM are endogenous and possess biological activity [4–7]. For example, 3,5-diiodothyronine (3, 5-T_2_) exerts thyromimetic action in rodents [8, 9] and treatment with 3-iodothyronamine (3-T_1_AM) or 4-[4-(2-aminoethylphenoxy)]phenol (T_0_AM) produces partially TH antagonistic effects such as hypothermia in mice and Djungarian hamsters [10, 11]. The mechanisms of action of TH and THM in cell culture systems *in vitro* are of high scientific interest; however, uptake, release and intracellular metabolism affect their bioavailability or may lead to the formation of products with their own distinct biological activity in the experimental system. To elucidate how TH and THM are utilised by cell types derived from different tissues can help clarify their mode(s) of action. Hence, the development of a validated and convenient analytical method for TH, TAM and TAc in cell extracts *in vitro* is of major importance.

We recently published a validated analytical method based on liquid-liquid extraction and isotope dilution high performance liquid chromatography/tandem mass spectrometry (LC-MS/MS) for the determination of 15 TH/THM (T_0_ to T_4_ thyronines (TN) and TAM, see Fig 1 for a complete list of compounds) in cell culture media extracts [12]. The method demonstrated the accurate, reproducible quantification of THM within a single 10 min analysis, with lower limits of quantification (LLOQ) in the range 0.078–0.234 nM. We applied the method to quantify the de-iodination metabolites 3,3’-T_2_, 3-T_1_ and T_0_ that were detected in DMEM medium when T_3_ was incubated with primary hepatocytes [12]. We recently reported a preliminary adaption of the above method to analyse a limited number of TN (T_4,_ T_3_ and rT_3_) in Madin-Darby canine kidney 1 cell lysate extracts as part of a study on molecular characterization of monocarboxylate transporters involved in cellular TH uptake and efflux [13].

We now describe the extension of this method to enable the analysis and quantification of TH, THM and TAc in extracted, lysed cells. The method has been validated for the 15 TH/THM, using eight inter day replicates, for the human hepatocellular carcinoma cell line Hep G2, applying US Federal Drug Administration guidelines [14]. In addition, the method was validated (with 3-inter day repeats) for rat thyroid epithelium PCCL3 cells [15, 16] with the inclusion of 3-iodothyroacetic acid (3-T_1_Ac) and thyroacetic acid (T_0_Ac). We applied the latter method to the elucidation of potential metabolism pathways in cultured rat thyroid PCCL3 cells by incubating with 3-T_1_, 3-T_1_AM and 3-T_1_Ac.

## Materials and methods

### Chemicals, reagents, buffers, calibrators and quality control samples

The internal standards (IS) ^13^C_6_-T_4_, ^13^C_6_-T_3_, ^13^C_6_-rT_3_, and ^13^C_6_-3,3’-T_2_ were obtained from Isosciences LLC (King of Prussia, PA, USA). Further IS, ^2^H_4_-3-T_1_AM, ^2^H_4_-3-T_1_Ac and ^2^H_4_-T_0_Ac, were a generous gift from Dr. T.S. Scanlan (Portland, OR, USA).

T_4_AM, T_3_AM and rT_3_AM, were provided by Dr. R. Thoma (Formula GmbH, Berlin, D), who also purified 3-T_1_AM by preparative HPLC. Dr. R. Smits (ABX Advanced Biochemical Compounds, Radeberg, Germany) performed synthesis of high-purity 3-T_1_AM and T₀AM. 3,5-T_2_AM, T_1_Ac and T0Ac were also kindly provided by T.S. Scanlan; T_4_, T_3_, 3,3′-T_2_, 3,5-T_2_, 3′,5′-T_2_, 3-T_1_, 3′-T_1_ and T₀ were sourced from Henning Arzneimittel GmbH (Berlin, Germany). ^13^C- and ^1^H-labelled internal standards contained no measurable concentrations of unlabelled analogues and no cross-contamination from other ^13^C- or ^1^H-labelled TH. TN, TAM and TAc were of minimum 99.6% purity. No corrections were made for potential cross-contaminating compounds.

Certified reference standards of T_3_, 3,3’-T_2_ and T_4_ at 100 μg/mL in 0.1 N ammonium hydroxide/methanol were purchased from Qmx Laboratories (Thaxted, UK).

CHROMASOLV® Plus 2-Propanol, *t*-butyl methyl ether, methanol and dimethyl sulfoxide (DMSO) were from Sigma-Aldrich Chemie GmbH (Munich, Germany) as was formic acid, hydrochloric acid (37%), glacial acetic acid, sodium hydroxide and EDTA. Sucrose was purchased from Carl Roth (Karlsruhe, Germany) and Hepes from Merck (Darmstadt, Germany). Water was purified by a Milli-Q water purification system from Millipore (Billerica, MA, USA) and was of 18.2 mΩ quality.

Coon’s F12 medium and fetal bovine serum (FBS) were purchased from Biochrom (Berlin, Germany); bovine TSH, insulin, transferrin, hydrocortisone, glycyl-histidyl-lysine somatostatin and DMEM (Dulbecco's Modified Eagle's medium) were obtained from Sigma Aldrich (Munich, Germany).

Individual stock solutions of TN, TAM and TAc were prepared at 10 mM in DMSO. A 20 μM mixed TN/TAM (15 compounds) working solution of T_4_, T_3_, rT_3_, 3,3′-T_2_, 3,5-T_2_, 3′,5′-T_2_, 3-T_1_, 3′-T_1_, T₀, T_4_AM, T_3_AM, rT_3_AM, 3,5-T_2_AM, 3-T_1_AM and T₀AM was prepared in 50% v/v methanol/water containing 0.1% formic acid (re-suspension buffer, RS). For PCCL3 analyses an additional mixed TN/TAM/TAc (17 compounds) working solution, also at 20 μM, was prepared containing 3-T_1_Ac and T_0_Ac in addition to the above THM. A working solution of certified reference standards containing a mixture of T_4_, T_3_ and 3,3′-T_2_ at 20 μM was prepared in RS. Further working solutions at 1000, 100 and 10 nM were prepared in RS and used to spike cell lysates to obtain calibrator and quality control samples. For Hep G2 cell lysates in 24-well cell culture plates, calibrators were spiked at 0.016, 0.031, 0.063, 0.125, 0.25, 0.5, 1.25, 2.5, 5, 20 and 50 nM. In-house and external spiked quality-control samples (QQAL) at low (LQAL, 0.10 nM), medium (MQAL, 12.5 nM) and high (HQAL, 40 nM) concentrations were prepared by spiking cell lysates with either the appropriate in-house or certified reference working standard solutions respectively. For PCCL3 cell lysates in 6-well cell culture plates, calibrators were spiked at 0.01, 0.05, 0.1, 0.5, 1.0, 2.5, 12.5, 25, 50, 125, 250 and 500 nM. In-house QQALs at low (LQAL, 0.10 nM), medium (MQAL, 1.0 nM) and high (HQAL, 2.5 nM) concentrations were prepared by spiking cell lysates with in-house working standard solutions.

IS stock solutions for Hep G2 analyses were prepared in DMSO and contained ^13^C_6_-T_4_, ^13^C_6_-T_3_, ^13^C_6_-rT_3_, ^13^C_6_-3,3′-T_2_ and ^2^H_4_-3-T_1_AM (all at 2 μM). For PCCL3 analyses an additional IS stock solution was prepared containing the same 5 stable-isotopically labelled standards plus ^2^H_4_-T_0_Ac and ^2^H_4_-T_1_Ac (also at 2 μM in DMSO). All stock solutions were stored at -20°C in brown glass vials with Teflon-lined screw tops.

### Cell culture

All cell culture work was performed under standard culturing conditions (5% CO_2_ and 37°C) with standard aseptic techniques used throughout. Hep G2 medium was used without phenol red indicator.

Hep G2 cells were cultured in DMEM culture medium supplemented with 10% FBS. Cells were seeded in 24-well plates and grown confluent. At 24 h post plating, the medium was changed. After a further 24 h the medium was again changed, but this time for culture media without FBS. This state of cellular starvation was maintained for another 24 h, after which plates destined for standard curve production were then taken to ice, the media aspirated and the wells washed twice with ice cold PBS. The plates were stored dry at -20°C.

Rat thyroid PCCL3 cells were cultured in Coon’s F12 medium supplemented with 5% FBS, 1 mU / ml bovine TSH and 5 hormones (10 μg / ml insulin, 5 μg / ml transferrin, 10 nM hydrocortisone, 20 ng / ml glycyl-histidyl-lysine and 10 ng / ml somatostatin). After seeding in 6-well plates at a density of 400,000 cells per well, the cells were grown for 24 h in medium as described above. 24 h before experiments the cells were washed twice with PBS and grown in medium without FBS to deplete them from TH and their metabolites. 24 h fasted PCCL3 cells that were to be used to investigate TH metabolism were subjected to incubations with TH (or just vehicle) in Krebs Ringer Buffer for various time points before being washed and stored in the same manner as those destined for use in making standard curves.

### Cell lysis and sample extraction

Homogenisation Buffer pH 7.4 (250 mM D-(+)Sucrose, 20 mM Hepes, 1 mM EDTA; HB) was made and stored in aliquots at -20°C for single use. HB was mixed 1:1 with 0.1 N NaOH to produce Lysis Buffer 1 (LB1). Lysis Buffer 2 (LB2) consisted of 30% v/v glacial acetic acid in HB. Both LB1 and LB2 were made freshly for each cell lysis.

The protocol stated was applied to both 6- (PCCL3) and 24- (Hep G2) well cell culture plates. Cells were lysed at room temperature by adding 200 μl LB1 per well with orbital shaking (600 rpm) for 3 min followed by the addition of 100 μl LB2. Calibrator and quality control designated wells were spiked with 95 μl of appropriate TN/TAM or TN/TAM/TAc working solution; those wells designated as unspiked or matrix controls received 95 μl RS. All wells except those designated as matrix controls were then spiked with 5 μl of the appropriate IS mix.

The resulting 400 μl cell lysates were transferred to 2.0 ml Eppendorf tubes and incubated in the dark at 37°C for 1 h. 1 ml of 30% v/v 2-propanol /*t*-butyl methyl ether was added to the lysate and the mixture was vortexed for 5 min. The phases were separated via centrifugation at 2000 rcf for 5 min, at room temperature. The upper (non-aqueous) phase was transferred to a new 2.0 ml Eppendorf tube, and the extraction process was repeated with a further 1 ml of 2-propanol /*t*-butyl methyl ether. The combined organic phases were dried to a pellet in an Eppendorf 5301 Concentrator.

Samples, calibrators and quality controls were re-suspended in 100 μl of RS. Samples designated as matrix controls were re-suspended in 95 μl of TN/TAM or TN/TAM/TAc working solution at the appropriate concentration, and then spiked with 5 μl of the appropriate IS Mix. All samples were stored at -20°C for LC-MS/MS analysis.

### Incubation of PCCL3 cells with mono-iodinated TH, TAM and TAc

After 24 h under FBS depletion, PCCL3 cells were incubated +/- 500 nM 3-T_1_AM, 3-T_1_, 3-T_1_Ac or DMSO in Krebs Ringer buffer (119 mM NaCl, 4.74 mM KCl, 1.19 mM KH_2_PO_4_, 25 mM NaHCO_3_, 2.54 mM CaCl_2_, 1.19 mM MgCl_2_, 10mM Hepes, 11 mM glucose, pH 7.4, 1.5mL) for 5, 10 or 30 min. After removal of the incubation buffer, cells were washed 3 times with PBS and frozen at -20°C to await cell lysis and extraction as described in detail in the previous section. Supernatants were also stored at -20°C; 400 μL aliquots were extracted as previously described [12]and analysed with the LC-MS/MS method described in the next section.

### HPLC–MS/MS analysis

Sample analyses were performed by auto-injection of samples with a PAL HTC-xt auto-sampler (CTC Analytics AG, Zwingen, Switzerland) onto an HSS PFP 2.5 μm 3.0x100 mm column (Waters, Milford, MA, USA) maintained at 40°C and served by a 1260 binary HPLC system (Agilent technologies GmbH, Waldbronn, Germany). The HPLC column outlet was linked to a Sciex API 6500 QTRAP mass spectrometer (SCIEX Germany GmbH, Darmstadt, Germany) fitted with a Turbo Spray IonDrive electrospray source. The positive ion electrospray LC-MS/MS method used for the analysis of TN, TAM and accompanying IS has been described in detail [12] and was applied without modification to the analysis of extracted Hep G2 cell lysates.

For the analysis of extracted PCCL3 cell lysates the electrospray method was extended to facilitate positive/negative ion switching. The source temperature was reduced to 450°C and the ionspray voltage was -4500 V. The additional MRM transitions listed in Table 1 were incorporated into the negative ion experiment. Dwell times of 10 ms were used throughout. The complete duty cycle (positive plus negative ion) amounted to 0.73 sec.

**Table 1:** Negative ion electrospray tandem mass spectrometry and retention time (R_t_) parameters for thyroacetic acids and internal standards.

Compound	R_t_ (min)	PI (m/z)	Pro 1 (m/z)	Pro 2 (m/z)	DP (V)	CE (V)	CXP (V)
^**2**^**H**_**4**_**-T**_**0**_**Ac**	4.71	247.0	203.1	105.9	-15	-14 (-26)	-9 (-5)
^**2**^**H**_**4**_**-T**_**1**_**Ac**	5.15	372.9	329.0	126.8	-5	-8 (-16)	-15 (-13)
**T**_**0**_**Ac**	4.72	243.0	198.9	106	-10	-12 (-24)	-9 (-5)
**3-T**_**1**_**Ac**	5.16	369.0	324.9	126.7	-15	-8 (-26)	-13 (-19)

PI: precursor ion; Pro 1: quantifier product ion; Pro 2: confirmation product ion; DP: declustering potential; CE: collision energy; m/z: mass to charge ratio; CXP: collision exit potential; V: volts. Values in parentheses refer to Pro 2.

For both Hep G2 and PCCL3 experiments the relative recoveries (RE), process efficiencies (PE) and matrix effects (ME) were determined. The ME represents the impact of interfering compounds (from the cell lysates and buffers) on analyte ionisation [17] and was calculated as the percentage of [post extraction spiked MRM peak area]/[spiked solvent MRM peak area] [18]. The PE, representing the amount of substance recovered after undergoing the extraction process, is defined as the percentage of the [pre extraction spiked MRM peak area]/[post extraction spiked MRM peak area]. A poor PE thus indicated the analyte(s) of interest were lost during the extraction process [19]. The RE describes the percentage of the [pre extraction spiked MRM peak area]/[spiked solvent MRM peak area]. Lower limits of quantification (LLOQ) and detection (LLOD) were determined as described in reference [12] for calibrator sample chromatographic peaks passing precision and accuracy tolerances ≤20% and showing signal:noise >10:1 and >6:1 respectively.

## Results

### Chromatographic peak measurement and identification

**Fig 2:**
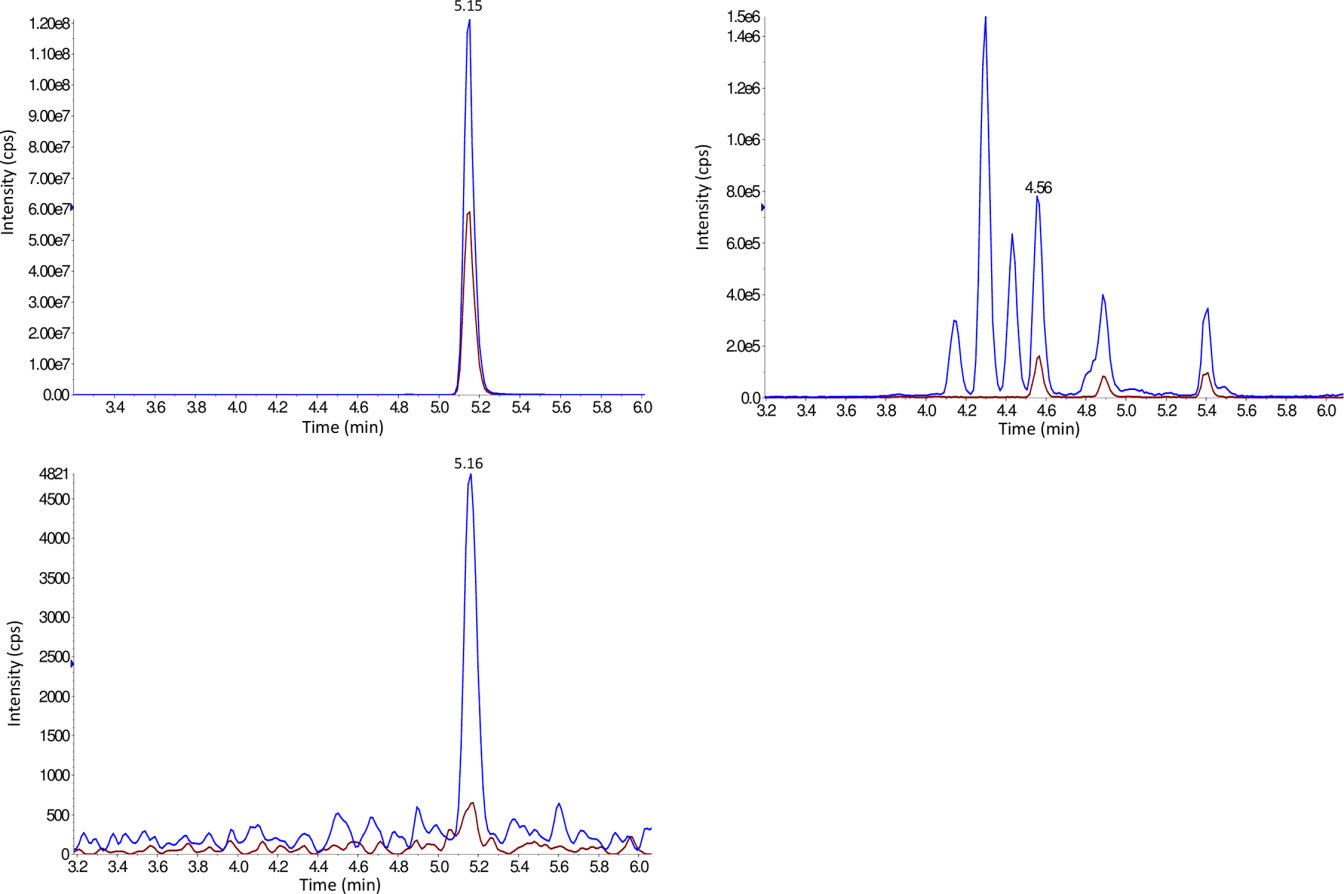
T_1_AM, T_0_AM and T_1_Ac chromatograms from a PCCL3 cell lysate extract after 5 min incubation with T_1_AM. 2 MRM Traces per analyte, overlayed. Upper trace: T_1_AM, middle trace: T_0_AM (4.56 min), lower trace: T_1_Ac.

**Fig 3:**
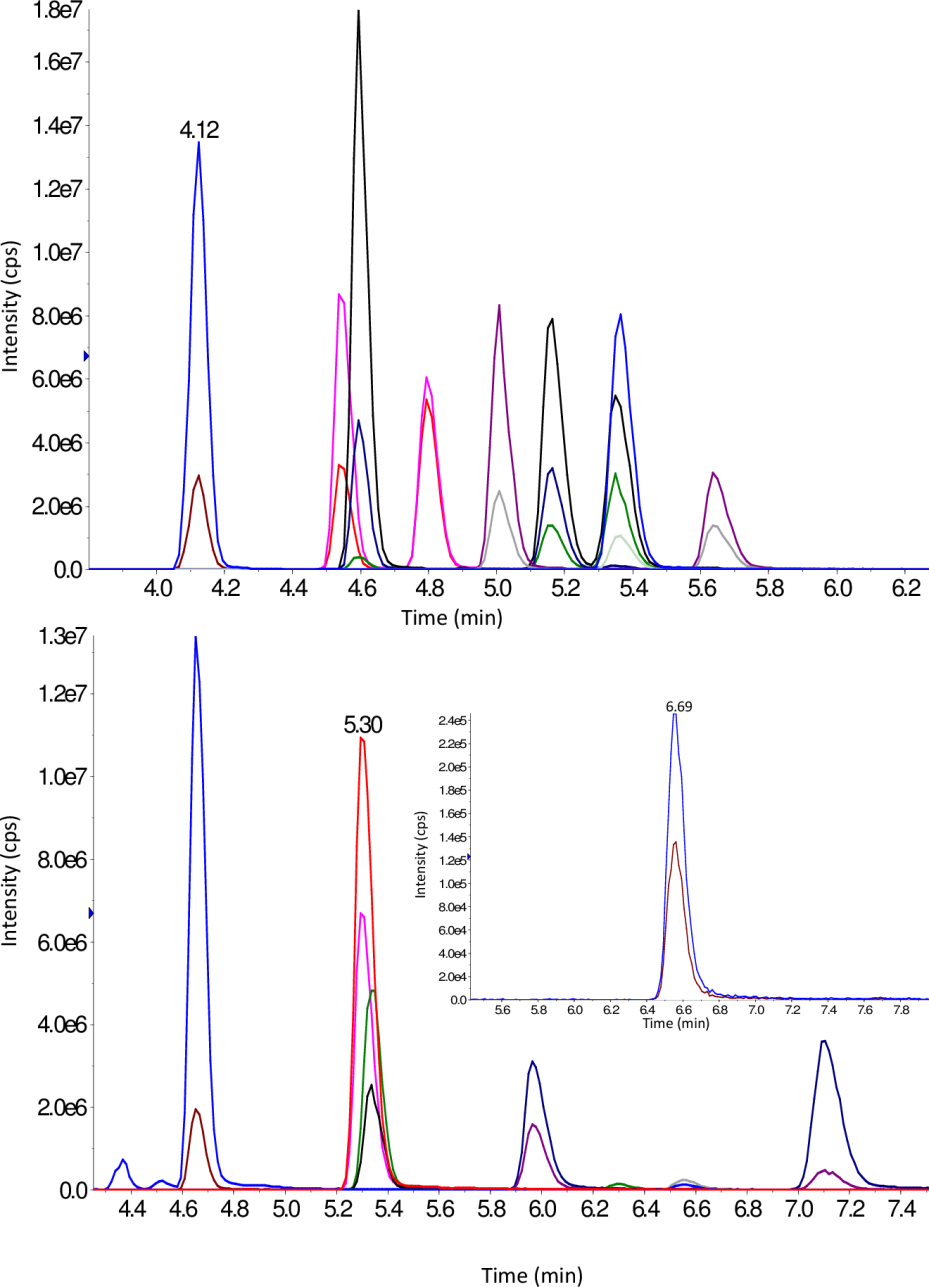
Thyronamine and thyronine chromatograms from Hep G2 cell lysate extracts spiked at 50 nM. 2 MRM Traces per analyte, overlayed. Upper trace: T_0_ (4.12 min); 3-T_1_ (4.54 min); 3-T_1_‘ (4.80 min); 3,5-T_2_ (4.60 min); 3,3‘-T_2_ (5.16 min); 3‘,5‘-T_2_ (5.35 min); T_3_ (5.01 min); rT_3_ (5.64 min); T_4_ (5.36 min). Lower trace: T_0_AM (4.65 min); T_1_AM (5.30 min); 3,5-T_2_AM (5.34 min); T_3_AM (5.97 min); rT_3_AM (7.10 min); T_4_AM (inset, 6.56 min).

Representative chromatograms for TAM and TN in Hep G2 and for TAM, TN and TAc in PCCL3 cell lysate extracts are displayed in Figs 2 and 3 respectively. For both Hep G2 and PCCL3 methods the lowest chromatographic peak width was 12 sec. (obtained for 3,3’-T_2_); the MS duty cycle allowed for a minimum of 15 data points to describe each peak to ensure reproducible peak integration. For each analyte and internal standard two MRM transitions were monitored; in most cases (except for 3-T_1_AM where background peaks interfered at the LLOQ) the most intense peak was used as quantification ion and the less intense peak as confirmation ion. To confirm the identity of metabolites arising from the incubation of PCCL3 cells with TH, the observed quantification ion/confirmation ion MRM peak area ratios (MRM_qc_) were compared with expected values (calculated from a standard solution of TN/TAM/TAc in RS) applying a ± 20% tolerance.

### Excellent linearity for most THM in both cell lines and acceptable LLOD/LLOQ

1/x Weighted linear regression calibration curves for each analyte were obtained by plotting MRM peak area ratios (analyte/IS) against concentration over the working range 0.016–50 nM for Hep G2 cell lysate extracts (except for T_0_: 0.50–50 nM) and 0.01–125 nM for PCCL3 cell lysate extracts. 1/x weighted linear correlation coefficients (r^2^) for all compounds are displayed in Table 2, and excellent linearity across the dynamic range of both methods for all analytes is evidenced by a minimum value for r^2^ of 0.994.

**Table 2:** Linearity and lower limits of detection and quantification for TH and TAM spiked lysed Hep G2 and PCCL3 cell extracts in 24- and 6-well plates respectively.

	Hep G2			PCCL3		
Compound	R^2^ Range	LLOQ [nM]	LLOD [nM]	R^2^ Range	LLOQ [nM]	LLOD [nM]
**T**_**0**_	0.995–0.999	*	*	0.996–1.000	0.050	0.025
**3-T**_**1**_	0.996–1.000	0.063	0.031	0.997–1.000	0.050	0.025
**3'-T**_**1**_	0.995–1.000	0.031	0.016	0.997–1.000	0.050	0.025
**3,5-T**_**2**_	0.996–1.000	0.125	0.063	0.996–1.000	0.050	0.025
**3,3'-T**_**2**_	0.996–1.000	0.063	0.031	0.998–0.999	0.100	0.025
**3',5'-T**_**2**_	0.995–0.999	0.031	0.016	0.997–1.000	0.050	0.025
**T**_**3**_	0.995–1.000	0.063	0.016	0.997–0.999	0.040	**
**rT**_**3**_	0.997–1.000	0.063	0.031	0.998–0.999	0.100	0.010
**T**_**4**_	0.996–1.000	0.063	0.016	0.997–0.999	0.040	**
**T**_**0**_**AM**	0.995–1.000	0.125	0.063	0.996–0.999	0.500	0.025
**3-T**_**1**_**AM**	0.996–1.000	0.031	0.016	0.997–0.999	0.100	0.020
**3,5-T**_**2**_**AM**	0.996–1.000	0.063	0.031	0.998–1.000	0.050	0.025
**T**_**3**_**AM**	0.994–1.000	0.063	0.031	0.997–0.999	0.050	0.010
**rT**_**3**_**AM**	0.995–0.999	0.063	0.031	0.997–0.999	0.050	0.010
**T**_**4**_**AM**	0.996–0.998	0.250	0.125	0.995–0.999	0.500	0.100
**T**_**0**_**Ac**	-	-	-	0.995–0.999	1.000	0.330
**3-T**_**1**_**Ac**	-	-	-	0.996–0.999	1.000	0.600

R^2^: Pearson coefficient, 1/x weighted linear regression. LLOQ: lower limit of quantification LLOD: lower limit of detection.

*LLOD/LLOQ not quantifiable (lysed cell extracts contained ca. 0.10 nM T_0_)

** LLOD not quantifiable (lysed cell extracts contained ca. 0.010nM T_4_ and T_3_)

The LLOD were in the range of 0.016–0.125 nM for Hep G2 cell lysate extracts in 24-well format and 0.01–0.6 nM for PCCL3 cell lysate extracts in 6-well format. The LLOQ ranged from 0.031–0.250 nM for Hep G2 and 0.04–1.0 nM for PCCL3. LLOD and LLOQ could not be determined for T_0_ in Hep G2 cell lysate extracts, due to a background concentration of ca. 0.06 nM. Similarly, LLOD could not be calculated for T_3_ and T_4_ in PCCL3 cell lysate extracts because of ca. 0.01 nM background concentration for both TN.

### Reasonable accuracy and precision for spiked quality-control samples (QQAL) in both cell lines

For Hep G2 inter- and intra-batch precision and accuracy were determined at low (LQAL), medium (MQAL) and high (HQAL) concentrations of TN and TAM. Certified reference solutions of three TN (3,3’-T_2_, T_3_ and T_4_) were purchased from a commercial supplier, diluted accordingly and used alongside in-house prepared solutions for independent comparison. 8-Day inter-batch CVs for in-house QQALs (Table 3) were all < 13.5% across the entire concentration range, with the exception of T_0_ (not quantifiable for LQAL, otherwise < 5.7%), 3,5-T_2_, T_0_AM and T_4_AM (LLOQ < LQAL, otherwise < 12.5%) and < 8.7% for certified reference QQALs (with the exception of LQAL for 3,3’-T_2_ and T_3_ which were < 17.8%). Intra-batch CVs were < 15.0% (in-house solutions only, Table 4). 8-Day inter-batch accuracy for in house QQALs were between 92.5–109.4% and for certified reference QQALs between 94.0–114.5% (Table 3). Intra-batch accuracies were determined for in-house solutions only and exhibited values in the range 92.0–120.0% (Table 4).

**Table 3:** Inter-day (n = 8) accuracy and precision for TH and TAM spiked lysed Hep G2 cell extracts in 24-well plates.

Compound	LQAL 0.10 nM*				MQAL 12.5 nM*			HQAL 40.0 nM*		
	Mean conc. [nM]		CV [%]	Accuracy [%]	Mean conc. [nM]	CV [%]	Accuracy [%]	Mean conc. [nM]	CV [%]	Accuracy [%]
**T**_**0**_	-		-	-	12.4	4.2	99	40.6	5.7	102
**3-T**_**1**_	0.10		9.9	104	12.5	8.3	100	41.1	5.7	103
**3'-T**_**1**_	0.11		8.7	107	12.6	5.9	100	41.1	7.1	103
**3,5-T**_**2**_	<LLOQ				12.6	7.2	101	41.9	6.8	105
**3,3'-T**_**2**_	0.11 [0.11]		8.7 [14.2]	109 [114]	12.6 [13.2]	6.6 [7.2]	100 [106]	41.5 [41.5]	6.9 [6.6]	104 [104]
**3',5'-T**_**2**_	0.11		10.8	106	12.6	5.6	101	40.8	5.5	102
**T**_**3**_	0.11 [0.09]		9.3 [17.8]	105 [94]	12.4 [11.8]	8.3 [8.7]	99 [95]	42.8 [39.4]	7. [6.0]	107 [99]
**rT**_**3**_	0.10		7.5	104	12.3	8.2	98	41.0	6.9	102
**T**_**4**_	0.09 [0.10]		13.5 [8.1]	108 [103]	9.8 [12.1]	8.3 [6.1]	98 [96]	35.0 [39.4]	7.1 [5.0]	109 [98]
**T**_**0**_**AM**	<LLOQ				12.8	5.7	102	41.6	6.5	104
**3-T**_**1**_**AM**	0.11		12.0	107	12.5	6.4	100	41.3	5.7	103
**3,5-T**_**2**_**AM**	0.10		6.1	103	12.0	5.8	96	41.3	5.8	103
**T**_**3**_**AM**	0.10		10.1	100	12.3	8.6	98	41.0	8.4	102
**rT**_**3**_**AM**	0.10		11.4	104	12.1	11.1	97	41.2	8.8	103
**T**_**4**_**AM**	<LLOQ				11.6	12.5	92	40.1	11.0	100

CV [%]: Percentage coefficient of variation. LQAL: low concentration spiked quality-control sample. MQAL: medium concentration spiked quality-control sample. HQAL: high concentration spiked quality-control sample. Values in parentheses were obtained by spiking samples with externally purchased TH standard solutions (see experimental section); all other values were from internally prepared TH standard solutions. Lysed cell extracts contained ca. 0.060 nM T_0_ making quantification at 0.1 nM unreliable.

*All spiked concentrations for internally prepared TH standard solutions as labelled except: T_4_ LQAL (0.08 nM), MQAL (10 nM), HQAL (32 nM).

**Table 4:** Intra-day (n = 8) accuracy and precision for TH and TAM spiked lysed Hep G2 cell extracts in 24-well plates.

	LQAL 0.10 nM			MQAL 12.5 nM			HQAL 40.0 nM		
Compound	Mean conc. [nM]	CV [%]	Accuracy [%]	Mean conc. [nM]	CV [%]	Accuracy [%]	Mean conc. [nM]	CV [%]	Accuracy [%]
**T**_**0**_	-	-	-	12.1	6.9	97	38.9	6.4	97
**3-T**_**1**_	0.10	12.6	104	12.6	5.7	101	40.4	5.5	101
**3'-T**_**1**_	0.09	15.0	92	12.1	6.3	97	40.0	6.6	100
**3,5-T**_**2**_	<LLOQ			12.5	5.8	100	40.0	5.4	100
**3,3'-T**_**2**_	0.10	8.5	99	12.3	5.0	99	39.5	4.8	99
**3',5'-T**_**2**_	0.10	11.3	95	12.3	5.3	98	40.2	5.9	100
**T**_**3**_	0.10	10.2	104	12.2	5.1	98	38.7	7.6	97
**rT**_**3**_	0.12	11.9	116	11.5	3.1	92	38.2	8.0	96
**T**_**4**_	0.11	12.8	108	12.0	4.5	96	39.9	5.1	100
**T**_**0**_**AM**	<LLOQ			12.2	5.1	98	38.7	7.6	97
**3-T**_**1**_**AM**	0.11	9.6	108	12.5	5.4	100	40.1	6.1	100
**3,5-T**_**2**_**AM**	0.12	6.3	120	12.2	6.1	97	40.7	5.1	102
**T**_**3**_**AM**	0.12	11.2	116	11.9	6.5	95	39.6	7.3	99
**rT**_**3**_**AM**	0.11	9.7	110	11.9	5.7	95	38.9	7.8	97
**T**_**4**_**AM**	<LLOQ			12.9	4.3	103	38.0	7.6	95

Lysed cell extracts contained ca. 0.10 nM T_0_ making quantification at 0.10 nM unreliable.

For PCCL3 inter-batch precision and accuracy were determined for QQALs using in-house prepared spike-solutions at 3 concentrations spanning the appropriate range for the occurrence of THM investigated in the accompanying application (0.10, 1.0 and 2.5 nM). 3-Day inter-batch CVs were all < 10.5% with the exception of the T_2_, T_3_ and T_4_ TAM (< 23.1% for LQAL, < 14.2% for MQAL and < 16.4% for HQAL) (Table 5). Intra-batch CVs (Table 6), measured at 0.10 (LQAL), 12.5 (MQAL) and 40 (HQAL) nM were < 6.1% for all THM for HQAL, < 4.4% at MQAL (with the exception of rT_3_AM, T_3_AM and T_4_AM which were below 19.4%) and < 9.1% at LQAL (with the exception of 3-T_1_, 3’-T_1_ and T_0_AM, which were < 14%). 3-Day inter-batch accuracy for all QQALs (Table 5) were between 92.6–118.7% for all TN and TAM, with the exception of T_0_AM, T_4_AM, T_0_Ac and T_1_Ac for LQAL which was <LLOQ for these compounds. Intra-batch accuracies for all QQAL were in the range 88.7–108.8% (Table 6). The necessity for using weighted linear regression for calibration curves that span a large concentration range was evidenced by comparison of the mean accuracy value obtained at the LQAL (0.1 nM) concentration for PCCL3 cells with or without 1/x weighting across a range of TN/TAM (14 compounds) for a single day. The mean % error using 1/x weighted calibration curves was 12%, whereas for non-weighted curves the mean % error was 233%.

**Table 5:** Inter-day (n = 3) accuracy and precision for TH and TAM spiked lysed PCCL3 cell extracts in 6 well plates.

	LQAL 0.10 nM			MQAL 1.0 nM			HQAL 2.5 nM		
Compound	Mean Conc. [nM]	CV [%]	Accuracy [%]	Mean Conc. [nM]	CV [%]	Accuracy [%]	Mean Conc. [nM]	CV [%]	Accuracy [%]
**T**_**0**_	0.11	14.8	111	1.1	5.0	107	2.7	3.6	107
**3-T**_**1**_	0.11	9.6	112	1.1	1.9	105	2.7	1.5	107
**3'-T**_**1**_	0.11	7.0	106	1.0	1.4	104	2.6	1.6	105
**3,5-T**_**2**_	0.10	5.3	103	1.1	1.9	111	2.8	1.5	113
**3,3-'T**_**2**_	0.11	10.4	106	1.0	2.3	104	2.6	1.6	106
**3',5-'T**_**2**_	0.11	10.5	106	1.0	4.9	104	2.7	5.0	108
**T**_**3**_	0.10	1.7	96	1.0	2.6	100	2.6	2.6	106
**rT**_**3**_	0.11	10.1	107	1.0	1.8	100	2.6	1.6	104
**T**_**4**_	0.11	10.4	108	1.0	3.2	100	2.6	2.0	104
**T**_**0**_**AM**	<LLOQ			1.0	7.9	101	2.6	7.2	106
**3-T**_**1**_**AM**	0.11	8.1	111	1.0	1.9	105	2.7	1.6	109
**3,5-T**_**2**_**AM**	0.11	13.1	110	1.0	3.1	102	2.7	4.0	108
**T**_**3**_**AM**	0.12	21.8	119	1.0	9.1	95	2.6	9.1	104
**rT**_**3**_**AM**	0.11	23.1	107	0.9	13.2	93	2.6	13.8	102
**T**_**4**_**AM**	<LLOQ			0.9	14.2	87	2.4	16.4	95
**T**_**0**_**Ac**	<LLOQ			0.9	5.9	90	2.7	2.5	107
**3-T**_**1**_**Ac**	<LLOQ			1.0	1.8	101	2.5	1.6	100

**Table 6:** Intra-day (n = 8) accuracy and precision for TH, TAM and TAc in spiked lysed PCCL3 cell extracts in 6-well plates.

	0.10			12.5			40.0		
Compound	Mean Calculated Concentration	% Relative Standard Deviation	% Accuracy	Mean Calculated Concentration	% Relative Standard Deviation	% Accuracy	Mean Calculated Concentration	% Relative Standard Deviation	% Accuracy
T0	0.10	8.6	103	12.7	3.8	102	39.8	1.4	99
3T1	0.10	12.6	99	12.5	3.2	100	39.3	2.2	98
3'T1	0.09	12.7	94	12.8	2.8	103	40.1	1.1	100
3,5T2	0.10	5.3	100	12.9	2.4	103	39.3	2.2	98
3,3'T2	0.10	9.1	98	12.8	1.2	102	40.5	1.3	101
3',5'-T2	0.11	6.6	108	12.6	2.4	101	39.9	1.9	100
T3	0.11	8.6	106	12.7	1.9	102	40.4	1.8	101
rT3	0.11	7.0	106	12.2	1.5	97	38.7	3.6	97
T4	0.10	4.5	103	12.6	1.6	101	39.9	1.6	100
T0AM	0.09	14.0	89	12.5	1.2	100	39.0	1.7	98
3T1AM	0.10	5.6	95	12.4	0.8	99	39.9	1.0	100
3,5T2AM	0.09	7.9	94	12.1	3.2	97	39.5	1.5	99
T3AM	0.10	8.8	104	11.1	17.0	89	39.4	3.2	98
rT3AM	0.10	6.3	101	11.1	18.8	89	38.4	4.2	96
T4AM	<LLOQ			11.4	19.4	91	36.9	6.1	92
T0Ac	<LLOQ			12.6	2.5	101	39.3	3.5	98
T1Ac	<LLOQ			13.6	4.4	109	40.2	3.7	101

### Recoveries, matrix effects and process efficiencies as analysed in two cell lines

Recoveries, process efficiencies and matrix effects determined for extracted Hep G2 cell lysates in 24-well plate format are displayed in Table 7. The recoveries for all TN, TAM and IS across the range of QQALs (0.1 to 40 nM) were between 73–93%, the process efficiencies between 51–95%, and the matrix effects between 70–106%.

**Table 7:** Inter-day (n = 8) process efficiency, matrix effect and recovery for TH and TAM spiked lysed Hep G2 cell extracts in 24-well plates.

Compound	0.10 nM									12.5 nM									40.0 nM								
	PE	±	%RSD	ME	±	%RSD	RE	±	%RSD	PE	±	%RSD	ME	±	%RSD	RE	±	%RSD	PE	±	%RSD	ME	±	%RSD	RE	±	%RSD
^**2**^**H**_**4**_**-3-T**_**1**_**AM**	69	±	12	84	±	11	82	±	10	69	±	10	82	±	9	81	±	13	71	±	10	86	±	6	83	±	13
^**13**^**C**_**6**_**-3,3'T**_**2**_	73	±	8	87	±	8	85	±	8	74	±	8	83	±	5	85	±	11	75	±	9	87	±	6	86	±	11
^**13**^**C**_**6**_**-T**_**3**_	78	±	8	91	±	7	86	±	8	80	±	8	88	±	5	89	±	11	78	±	10	88	±	8	88	±	12
^**13**^**C**_**6**_**-rT**_**3**_	67	±	10	84	±	11	80	±	7	69	±	8	79	±	8	85	±	10	70	±	10	84	±	8	84	±	12
^**13**^**C**_**6**_**-T**_**4**_	67	±	13	84	±	13	80	±	10	69	±	11	80	±	10	84	±	11	68	±	10	81	±	10	85	±	12
**T**_**0**_	148	±	17	170	±	15	87	±	9	71	±	8	91	±	7	76	±	12	72	±	11	92	±	6	78	±	13
**3-T**_**1**_	78	±	10	85	±	8	91	±	8	74	±	10	88	±	8	83	±	13	75	±	10	90	±	7	84	±	11
**3'-T**_**1**_	74	±	9	84	±	12	88	±	10	70	±	9	84	±	7	81	±	10	73	±	10	87	±	7	84	±	13
**3,5-T**_**2**_	<LLOQ			<LLOQ			<LLOQ			81	±	10	96	±	8	83	±	12	80	±	11	94	±	6	86	±	10
**3,3'-T**_**2**_	75	±	9	82	±	10	92	±	10	73	±	9	87	±	6	84	±	10	76	±	13	88	±	6	86	±	11
**3',5-'T**_**2**_	70	±	13	80	±	13	88	±	11	67	±	9	83	±	9	81	±	11	71	±	10	86	±	7	82	±	11
**T**_**3**_	81	±	13	89	±	10	91	±	10	72	±	11	87	±	8	81	±	12	75	±	11	90	±	7	84	±	11
**rT**_**3**_	75	±	10	86	±	11	88	±	10	68	±	8	85	±	7	83	±	10	71	±	10	87	±	6	82	±	11
**T**_**4**_	72	±	20	83	±	18	86	±	12	64	±	15	83	±	13	79	±	10	68	±	15	86	±	9	79	±	12
**T**_**0**_**AM**	<LLOQ			<LLOQ			<LLOQ			74	±	13	87	±	9	83	±	13	77	±	13	90	±	7	84	±	12
**3-T**_**1**_**AM**	75	±	13	87	±	15	87	±	14	69	±	11	87	±	8	78	±	13	72	±	12	90	±	5	80	±	13
**3,5-T**_**2**_**AM**	71	±	13	84	±	12	85	±	14	64	±	14	85	±	11	74	±	12	67	±	14	86	±	8	78	±	15
**T**_**3**_**AM**	64	±	26	77	±	33	85	±	19	61	±	15	79	±	16	80	±	11	66	±	13	84	±	9	78	±	16
**rT**_**3**_**AM**	63	±	14	80	±	14	79	±	19	59	±	15	79	±	11	79	±	11	62	±	16	81	±	8	76	±	18
**T**_**4**_**AM**	<LLOQ			<LLOQ			<LLOQ			51	±	21	70	±	11	79	±	10	60	±	21	74	±	15	73	±	24

Values were obtained by spiking samples with in-house prepared TH standard solutions. Lysed cell extracts contained ca. 0.10 nM T_0_, thus results at 0.10 nM are skewed.

The same QA parameters were determined at a single concentration (1 nM) for extracted PCCL3 cell lysates in 6-well plate format. Recoveries for all TN, TAM and TAc, including IS varied from 75–101%, with % CV that varied between 2 and 17%. Process efficiencies were in the range 58–89% with % CV from 1–17%; matrix effects ranged from 77–98% with % CV between 1 and 14%. This data is displayed in Table 8.

**Table 8:** Inter-day (n = 3) process efficiency, matrix effect and recovery for TH and TAM spiked lysed PCCL3 cell extracts in 6 well plates.

Compound	QQAL 1 nM (n = 3)								
	PE	±	SD	ME	±	SD	RE	±	SD
^**2**^**H**_**4**_**-T**_**0**_**Ac**	89	±	12	89	±	8	100	±	17
^**2**^**H**_**4**_**-T**_**1**_**Ac**	80	±	7	98	±	7	82	±	6
^**2**^**H**_**4**_**-3-T**_**1**_**AM**	76	±	6	90	±	7	85	±	7
^**13**^**C**_**6**_**-3,3'T**_**2**_	77	±	5	94	±	7	82	±	4
^**13**^**C**_**6**_**-T**_**3**_	80	±	6	93	±	3	85	±	7
^**13**^**C**_**6**_**-rT**_**3**_	66	±	7	88	±	6	75	±	6
^**13**^**C**_**6**_**-T**_**4**_	72	±	10	92	±	4	79	±	11
**T**_**0**_	72	±	3	87	±	2	82	±	3
**3-T**_**1**_	81	±	5	90	±	3	90	±	3
**3'-T**_**1**_	78	±	6	93	±	3	84	±	4
**3,5-T**_**2**_	88	±	9	96	±	4	91	±	6
**3,3-T**_**2**_	79	±	4	93	±	3	85	±	2
**3',5'-T**_**2**_	75	±	6	94	±	1	80	±	7
**T**_**3**_	74	±	2	91	±	2	81	±	4
**rT**_**3**_	70	±	5	89	±	4	79	±	5
**T**_**4**_	68	±	10	94	±	3	78	±	3
**T**_**0**_**AM**	84	±	8	97	±	2	87	±	8
**3-T**_**1**_**AM**	79	±	4	90	±	1	88	±	5
**3,5-T**_**2**_**AM**	73	±	5	88	±	3	83	±	3
**T**_**3**_**AM**	69	±	1	82	±	11	84	±	9
**rT**_**3**_**AM**	64	±	4	81	±	10	80	±	10
**T**_**4**_**AM**	58	±	10	79	±	13	80	±	14
**T**_**0**_**Ac**	77	±	17	77	±	14	101	±	12
**3-T**_**1**_**Ac**	82	±	8	96	±	13	86	±	5

### Application

#### Identification of TN, TAM and TAc metabolites generated by PCCL3 cells

Identification of THM in extracts of lysed PCCL3 cells and supernatants was confirmed or rejected by both R_t_ and MRM_qc_ as follows: T_0_: confirmed (R_t_ expected: 4.13, found: 4.10 min; MRM_qc_ within -4/+7% and -5/+14% of expected values in cell lysates and supernatants respectively); T_0_AM: confirmed (R_t_ expected: 4.51, found: 4.51 min; MRM_qc_ within -9/+4% and -14/+12% of expected values in cell lysates and supernatants respectively). 3-T_1_Ac: confirmed in cell lysates (Rt expected: 5.14, found: 5.13 min; MRM_qc_ within -14/+16% of expected values); confirmed only in cell supernatants with > 10 min incubation time (MRM_qc_ -11/+15% of expected values, for incubation times < 10 min the MRM_qc_ exceeded +/- 20%). T_0_Ac: unconfirmed in cell lysates (R_t_ expected: 4.71, found: 4.73 min; MRM_qc_ irreproducible and in many cases > ±20% of expected value); confirmed in supernatants with > 10 min incubation time (MRM_qc_ -18/+3% of expected values)).

#### Method application to cell extracts and supernatants of rat thyrocytes after incubation with mono-iodinated thyroid hormone metabolites

**Fig 4:**
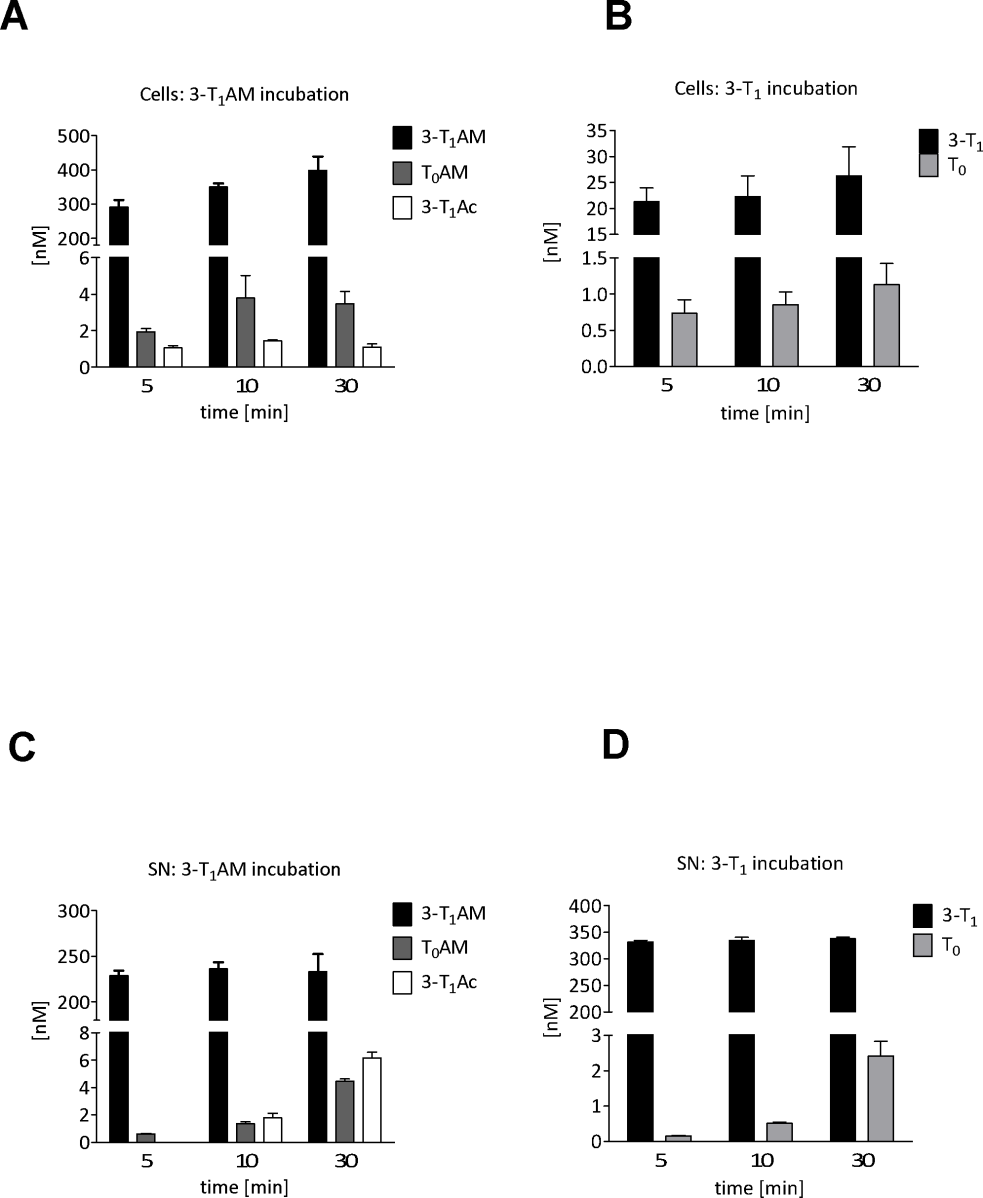
Application of the LC-MS/MS method to extracts of PCCL3 rat thyrocytes. THM in extracts of PCCL3 (A, B) cell and supernatants (C, D) after incubation with 500 nM 3-T_1_AM (A, C) or 3-T_1_ (B, D) in Krebs-Ringer buffer as measured by LC-MS/MS. Bars represent means of 3 independent experiments carried out on different days +/- SEM.

PCCL3 thyrocytes were incubated with 500 nM of mono-iodinated representatives of the TAM, the TN and the TAc family for 5, 10 and 30 min. After incubation with 3-T_1_AM the un-metabolised parent substance was detectable in cell lysates with a clear increase in concentration over time (Fig 4A) indicating its cellular uptake and intracellular accumulation. The extracellular concentration of 3-T_1_AM remained almost constant over the course of 30 min (Fig 4C). The de-iodination product T_0_AM and the de-amination product 3-T_1_Ac were detected in all cell extract samples (Fig 4A), and were below LLOD in DMSO treated negative controls and in the parent stock solution. These metabolites accumulated in the supernatants as evidenced (Fig 4C) by their increase in concentration over time. 3-T_1_ was detectable in cell lysates after incubation, indicating cellular uptake or binding of the parent substance (Fig 4B). Compared to 3-T_1_AM incubation, binding or uptake of 3-T_1_ occurred to a substantially lesser extent. Furthermore, in contrast to 3-T_1_AM, no clear increase in 3-T_1_ concentration was observed with incubation time in cell lysates. The T1 concentration in supernatants remained almost constant (Fig 4D).

The de-iodination product T_0_ was detected at low concentrations in all lysates after incubation with 3-T_1_ and in low but moderately increasing concentration in cell supernatants (Fig 4D), but not in DMSO controls or the 3-T_1_ stock solution. After incubation with 3-T_1_Ac, only small amounts of the parent substance were detectable in cell lysates for each time point (highest concentration approximately 5 nM, data not shown), the bulk of the 3-T_1_Ac remained unchanged in the cell supernatant (data not shown). As mentioned above, the putative de-iodination product T_0_Ac was detected in cell lysates, but (even after 30 min incubation time) at concentrations close to the limit of quantification making the confirmation of its identity irreproducible. However, T_0_Ac was detected in supernatants after 10 and 30 min incubation at mean concentrations of 5.6 and 10 nM respectively (data not shown).

Table 9 shows mass balance data for the above incubation experiments. For all three incubation substances over all time points, mass balance totals were in the range of 66–84%. For 3-T_1_ incubation, 4–5% of the parent substance was accounted for in the extracts of cell lysates, 62–63% was evident in the supernatant. The de-iodinated metabolite T_0_ accounted for only 0.2% of the total mass balance in cell lysates after 30 min and in marginally higher amounts (0.4%) in supernatants. The percentage balance of 3-T_1_AM remained constant at ca. 40% in supernatants over the entire course of 3-T_1_AM incubation, but increased from 29% after 5 min to 41% after 30 min in cell lysates. The percentage balance of the de-iodination metabolite T_0_AM, which was present at low concentrations, increased in supernatants from 0.1 (10 min) to 0.8% (30min), but stayed constant at 0.2–0.4% in cell lysates. The de-amination product 3-T_1_Ac constituted only 0.1–0.2% of the total in cell lysates for all time points and was detected only after 30 min in supernatants at 1.2%. Finally, T_1_Ac was not substantially accumulated by PCCL3 cells and remained between 77–81% of the total accountable pmol amount in supernatants. The % pmol of the de-iodinated product T_0_Ac increased from 1.1 to 1.9% of total pmol from 10 to 30 min.

**Table 9:** Mass balance in cells/supernatants for THM after incubation of mono-iodinated THM with PCCL3 rat thyrocytes.

	Incubation time	5min							10min							30min						
Incubation compound (pmol)	Compound	Cells			Supernatants			Total	Cells			Supernatants			Total	Cells			Supernatants			Total
		pmol	(%)	|	pmol	(%)	|	pmol %	pmol	(%)	|	pmol	(%)	|	pmol %	pmol	(%)	|	pmol	(%)	|	pmol %
**T**_**1**_ **(775.9)**	**T**_**1**_	32	(4%)	|	498	(62%)	|	66%	34	(4%)	|	502	(63%)	|	67%	39	(5%)	|	507	(63%)	|	69%
	**T**_**0**_	1	(0.1%)	|	0.3	(0.03%)	|		1	(0.2%)	|	1	(0.1%)	|		2	(0.2%)	|	3	(0.4%)	|	
**3-T**_**1**_**AM (812.7)**	**3-T**_**1**_**AM**	238	(29%)	|	323	(40%)	|	69%	276	(34%)	|	333	(41%)	|	76%	334	(41%)	|	329	(40%)	|	84%
	**T**_**0**_**AM**	1	(0.2%)	|	1	(0.1%)	|		3	(0.4%)	|	2	(0.2%)	|		3	(0.3%)	|	6	(0.8%)	|	
	**3-T**_**1**_**Ac**	1	(0.1%)	|	nd	(0%)	|		1	(0.2%)	|	nd	(0%)	|		1	(0.1%)	|	9	(1.2%)	|	
**3-T**_**1**_**Ac (777.6)**	**3-T**_**1**_**Ac**	4	(0.6%)	|	596	(77%)	|	77%	4	(0.5%)	|	599	(77%)	|	79%	3	(0.3%)	|	632	(81%)	|	84%
	**T**_**0**_**Ac**	1	(0.1%)	|	nd	(0%)	|		1	(0.2%)	|	8	(1.1%)	|		1	(0.1%)	|	14	(1.9%)	|	

**pmol**: absolute amount in cell lysate (0.4 mL) or supernatant (1.5 mL), **(%)**: relative % pmol for individual compound with respect to pmol of incubation compound, **Total pmol %:** summation of % pmol for all compounds detected in cell lysates or supernatants with respect to pmol of incubation compound. % pmol values were not adjusted to account for relative recoveries.

## Discussion

### LC-MS/MS method validation

We applied our previously published LC-MS/MS method [12], with minor modifications, to the analysis of Hep G2 cell lysate extracts. In brief, the method features a 10 min analysis time for the 9 TN and 6 TAM in positive ion mode and complete baseline chromatographic separation for the three isobaric T_2_ isomers (due to identical precursor and product ion selections, these isomers must be separated chromatographically for individual quantification). Wherever possible, structurally identical, stable isotopically labelled IS for quantification were used for each analyte to enhance the accuracy of the method [20]. In cases where no identical IS was commercially available, an IS with the closest structural similarity was applied.

We also adapted this method for the analysis of PCCL3 cell lysate extracts by using positive/negative electrospray ionisation switching to further incorporate negative ion electrospray MRM transitions for T_0_Ac and T_1_Ac and their respective (^2^H_4_-T_0_Ac and ^2^H_4_-T_1_Ac) IS.

Across the range of QQAL concentrations and cell lines PE were ca. 17–24% lower than for those measured in cell culture media [12]. For the most part RE were not substantially worse for cell lysates extracts (4–13% less across both cell lines and QQAL concentrations) compared to culture media extracts. ME for Hep G2 in contrast to cell culture media tended to decrease substantially with increasing QQAL concentration and were 22% higher for the LQAL (0.10 nM) than for the culture media LQAL (0.75 nM), 15% higher at MQAL (10 vs 12.5 nM) but comparable at HQAL (40 vs 80 nM) concentrations. These results would seem to be consistent with greater LC-MS/MS ion suppression effects caused by the dirtier cell lysate matrix. The marginally lower RE could arise from hormone adhesion to cell culture plates.

### Application of the LC-MS/MS method to extracts of PCCL3 rat thyrocytes

We successfully applied the described LC-MS/MS method to investigate THM in PCCL3 thyrocytes after incubation with 3-T_1_AM, 3-T_1_ and 3-T_1_Ac. The data obtained demonstrate that 3-T_1_AM is de-iodinated to T_0_AM and de-aminated to 3-T_1_Ac, while 3-T_1_ is de-iodinated to T_0_ by PCCL3 cells. This indicates 3-T_1_AM and 3-T_1_ uptake and intracellular metabolism by the cell line and confirms tyrosyl ring de-iodination activity as well as activity of an amino oxidase in PCCL3 cells as previously suggested [21]. Thus, the interpretation of 3-T_1_AM effects on the hypothalamus-pituitary-thyroid (HPT) axis in mice treated with this biogenic amine [21] and actions observed in thyrocyte cell culture, should consider the possibility of direct biological action of its metabolites. Our findings of in vitro 3-T_1_AM de-iodination to T_0_AM and de-amination to 3-T_1_Ac are in line with previous data obtained in different cell lines by others ([22], [23]). However, in the related thyrocyte model FRTL-5 the catabolite T_0_Ac was detected additionally. In this study, virtually all of the produced T_0_Ac was detected in the incubation medium [22].

PCCL3 cells accumulate T_1_ and T_1_AM and T_0_AM, but not T_1_Ac or T_0_Ac. We observed a time-dependent increase in recovery, probably due to cellular accumulation of TH/THM, which may indicate some adhesion of TH/THM to cell culture plastic in the absence of TH/THM binding proteins in incubation medium. Cells de-iodinate T_1_ to T_0_, which mainly accumulates in the supernatant, suggesting uptake of T_1_ and export of T_0_. Cells also accumulate T_1_AM and de-iodinate it to T_0_AM, the majority of which is again exported; T_1_Ac also appears in supernatants, but only at later time points. In contrast, T_1_Ac is not accumulated by PCCL3 cells, but rather exported over time and also de-iodinated to T_0_Ac, which appears in the supernatant.

Generally, only limited metabolism of these TH/THM occurs in PCCL3 over the first 30 min. Less than 1% of T_1_ is de-iodinated to T_0_, around 5% of 3T_1_AM is metabolized to T_0_AM and 3T_1_Ac and less than 2.5% of 3T_1_Ac is de-iodinated to T_0_Ac when offered as the sole substrate to PCCl3 cells. Recoveries of TH and THM carrying only one or no iodine atom ranges between 66 and 84% (values were not corrected for any losses in the sample extraction process, the relative recoveries of the method would account for the fact that mass balances do not total 100%). These recoveries are comparable to those of the classical TH, T_4_ and T_3_, in this and other cell lines during uptake and metabolism experiments ([12], [13]). Interestingly, 3T_1_Ac exhibits the highest recovery and lowest accumulation in cell extracts, which might indicate that both its precursors 3T_1_ and 3T_1_AM are more avidly bound by cells, and metabolized intracellularly. Some (irreversible) binding of both substrates to cellular targets cannot be excluded. However, the time dependent increase in recovery may also indicate that initially these TH/THM compounds may absorb to cell culture plastic surfaces and thus escape complete extraction by our currently employed procedure.

The observation of significant amounts of iodine-free T_0_ in extracts of HepG2 cells cannot yet be explained satisfactorily. As blank solutions, reagents and standards used during the pre-analytical workup and LC-MS/MS analysis do not contain measurable amounts of T_0_, we suppose that it might be carried over from T_0_-containing FCS, which is required for the initial steps of cell culture. Alternatively, HepG2 cells may transiently accumulate T_0_ from serum in the culture medium or eventually generate it from accumulated TH and THM. Up to now, fate and metabolism of T_0_ have rarely been studied in cell culture experiments, whilst formation and renal secretion into urine has been reported using various methods ([24], [25], [26]). In humans, T_0_ has been described and quantified by immunoassays and mass spectrometry at low nanomolar concentrations in urine, indicating that not all iodothyronines are fully de-iodinated to recover their content of the essential trace element iodine for its recycling by the thyroid gland.

In conclusion, we have shown that TH and THM can be quantified precisely and accurately to a LLOQ of 0.031–0.125 nM for TN (except for T_0_ in HepG2 cells, which contained baseline concentrations) and 0.031–05 nM for TAM in Hep G2 and PCCL3 cell lysate extracts. ME for all TN, TAM and TAc were acceptable for both cell lines (>70%) and RE were >73% in all cases. For both cell lines, PE, an indication of the overall efficacy of pre-analytic extraction and LC-MS/MS measurement for TH and THM in the sample matrix, were substantially less than for culture media [12]. This is due to increased ME from the cell sample matrix. PE were however reproducible for all TN, TAM and TAc in PCCL3 cells (10% CV or better) with the exception of T_0_Ac (17% CV) and better than 20% for all TN and TAM in Hep G2 cell lysate extracts with the exception of T_3_AM and T_4_AM (26 and 21% CV respectively). We therefore expect the reported methods to find wide applicability for the quantitative analysis of TH and THM in cell lysates.

By applying our positive/negative electrospray switched tandem LC-MS/MS method to the analysis of cell lysate and supernatant extracts recovered from the 30 min incubation of mono-iodinated TN, TAM and TAc with PCCL3 rat thyrocytes, we were able to follow the progress of limited de-iodination of T1, T1AM and T1Ac and the de-amination of T1AM to T1Ac.
